# Prevalence and costs of US pediatric hospitalizations, 2022

**DOI:** 10.1002/jhm.70272

**Published:** 2026-02-10

**Authors:** Anna J. Lytchakov, Nathan M. Money, Jennifer A. Hoffmann, Todd A. Florin, Kenneth A. Michelson, Sriram Ramgopal

**Affiliations:** ^1^ Northwestern University Feinberg School of Medicine Chicago Illinois USA; ^2^ Department of Pediatrics, Division of Pediatric Hospital Medicine Primary Children's Hospital Salt Lake City Utah USA; ^3^ Departments of Pediatrics and Medical Social Sciences, Division of Emergency Medicine, Ann & Robert H. Lurie Children's Hospital of Chicago Northwestern University Feinberg School of Medicine Chicago Illinois USA; ^4^ Department of Pediatrics, Division of Emergency Medicine, Ann & Robert H. Lurie Children's Hospital of Chicago Northwestern University Feinberg School of Medicine Chicago Illinois USA

## Abstract

**Background:**

Pediatric hospitalizations represent an evolving component of US healthcare utilization. The coronavirus disease 2019 (COVID‐19) pandemic hastened rising mental health visits and shrinking rural hospital capacity. Understanding contemporary patterns in pediatric hospitalizations is critical to inform health system planning and policy decisions.

**Objectives:**

To describe the most common and costly diagnoses among US pediatric hospitalizations in 2022, using 2016–2019 data to contextualize trends in admission volume and cost.

**Methods:**

We conducted a cross‐sectional analysis of nonlive birth admissions for children (<18 years) using the 2022 Kids' Inpatient Database, the largest US all‐payer pediatric inpatient data set, supplemented by an evaluation of volume trends from 2016 to 2019. We evaluated the most common and costly diagnoses in 2022 and evaluated trends in volumes and costs from the prior study years.

**Results:**

There were 1.78 million pediatric hospitalizations in 2016, 1.69 million in 2019, and 1.59 million in 2022, representing a 10.5% overall decline. In 2022, the most common diagnoses were bronchiolitis (7.0%), major depressive disorder (5.2%), and respiratory failure (5.0%). Mental health conditions (major depressive disorder, mood disorder, and suicide and self‐inflicted injury) comprised three of the 20 most frequent diagnoses. Inflation‐adjusted costs increased from $32.1 billion in 2016 to $35.9 billion in 2022. The costliest conditions in 2022 included respiratory failure, septicemia, and chemotherapy. Rural hospitals represented only 3.1% of admissions in 2022, down from 4.5% in 2016.

**Conclusions:**

Pediatric inpatient care is increasingly centralized in urban and children's hospitals. Respiratory and mental health conditions are among the common conditions requiring hospitalization. These findings highlight the need for stronger regional coordination to support access to pediatric care for these common conditions.

## INTRODUCTION

Approximately 2 million pediatric hospitalizations occur annually in the United States, representing a substantial burden on patients, families, and the healthcare system.^1^ Identifying cost drivers in pediatric care can guide resource planning, policy decisions, and research prioritization. The coronavirus disease 2019 (COVID‐19) pandemic dramatically disrupted patterns of pediatric healthcare utilization, exacerbating longstanding disparities and accelerating changes in where and how children receive care.^2^ The lingering and downstream effects of the pandemic emphasize the need to understand how pediatric inpatient patterns have evolved across recent years. Early pandemic‐related closures, infection surges, and delayed preventive visits have reshaped the clinical landscape.^3^, ^4^ Several studies have reported that pediatric hospitals across the United States reported sharp declines in admissions for respiratory illnesses and elective procedures, followed by surges in mental health‐related encounters and viral infections in later years.^2^, ^4^

These shifts in common pediatric conditions requiring hospitalization occurred alongside changes in the acute care landscape, with reductions in available pediatric beds and staff. These disproportionately affected rural and community hospitals that had already faced resource limitations before 2020. Between 2008 and 2022, nearly one‐third of pediatric inpatient units and one‐fifth of pediatric beds closed nationally, while adult inpatient capacity remained largely stable.^4^ These losses have been accompanied by declines in pediatric hospitalizations at rural and urban nonteaching hospitals, which decreased fourfold and sixfold, respectively, between 2009 and 2019.^5^ Additionally, care has become increasingly concentrated in freestanding children's hospitals and large urban centers.^5^ The proportion of children experiencing interfacility transfer increased from 6.1% in 2000 to 18.8% in 2019, highlighting a growing reliance on regional referral systems to meet inpatient needs.^6^ These trends have raised concerns about reduced local access and diminished system resilience during high‐volume periods, as seen during the 2022 viral respiratory surge.^7^

Despite the importance of these changes, recent analyses have largely focused on pandemic‐era data alone or on narrow subsets of conditions or hospitals.^8^ Previous national studies describing pediatric hospitalizations, including those published before 2019, provided foundational insights into cost drivers and hospital characteristics but are now outdated.^1^, ^6^ Few studies have incorporated postpandemic data to capture emerging trends in mental health utilization, regional centralization of pediatric care, and the evolving role of community hospitals. The use of standardized frameworks using nationally representative pediatric hospitalization data provide an opportunity to evaluate how common conditions and associated costs have changed over time and investigate how pediatric inpatient care in the United States changed from pre‐ to postpandemic periods. We therefore sought to describe the most common and costly diagnoses among US pediatric hospitalizations in 2022, and to examine how diagnoses, costs, and the location of inpatient care have evolved over time, using 2016–2019 data to contextualize longitudinal shifts leading into the postpandemic period.

## METHODS

### Data source

We analyzed data from the 2016, 2019, and 2022 Kids' Inpatient Database (KID). The KID is published by the Healthcare Cost and Utilization Project (HCUP), which provides administrative data abstracted from hospital discharge records. The primary analysis was a cross‐sectional evaluation of pediatric hospitalizations in 2022, supplemented by a longitudinal assessment of trends in volumes, diagnoses, and costs using 2016 and 2019 data. The KID is the largest publicly available all‐payer pediatric inpatient care database in the United States. This study was approved by the authors' Institutional Review Board and followed strengthening the reporting of observational studies in epidemiology reporting guidelines. Artificial intelligence was not used in the development of this manuscript.

### Inclusion and exclusion criteria

We included inpatient encounters for patients <18 years of age. As our focus was on acute care admissions, we excluded encounters for live births. This was determined either through a dedicated variable within KID (I10_HOSPBRTH) indicating a hospital birth, or through the presence of “live birth” in a primary or secondary diagnosis code position using the Pediatric Clinical Classification System (PECCS). We elected to use this approach given that it allowed us to maintain consistency in variable coding over the three studied KID datasets. We additionally excluded encounters with missing cost information.

### Data abstraction

We extracted data elements from each of the KID datasets to describe the study samples. Patient demographics included race and ethnicity, sex, and payor status. Hospital details included hospital location (rural, urban nonteaching, urban teaching, and freestanding children's hospital) and census region. Categories of rural, urban nonteaching, and urban hospitals were identified using a variable for hospital location (hosp_location). Children's hospitals are identified in KID based on a variable used to define hospital stratum in KID (kid_stratum). Encounter details included diagnosis, length of stay, disposition, and whether an emergency department was associated with the inpatient encounter. We used the PECCS to classify encounters by primary diagnosis. The PECCS framework organizes all international classification of diseases, tenth revision, clinical modification (ICD‐10‐CM) diagnosis codes into 834 clinically meaningful categories to identify specific pediatric conditions.^9^

To obtain costs, we multiplied the total charge data in KID by the hospital‐specific cost/charge ratio. We calculated costs using the hospital‐specific all‐payer inpatient cost‐to‐charge ratio or the group average if unavailable. For the years 2016 and 2019, we adjusted costs for inflation to present all costs to dollars in 2022, using data from the U.S. Bureau of Labor Statistics prices for medical care expenses.^10^ We determined the presence of medical complexity, both overall and, when present, by category of complexity, using encounter‐level diagnosis and procedure codes based on the version 3 complex chronic conditions classification system.^11^

### Analysis

We described characteristics for each year of the study sample, including identifying the total number of admissions and population demographics. For the year 2022, we additionally presented these data stratified by hospital location.

To characterize the highest prevalence and costly conditions of children admitted to the hospital, our period of interest was 2022, with prior years used to evaluate trends in costs over time. From the 2022 KID sample, we identified the most prevalent and costly conditions requiring hospitalization. Following procedures described by Kaiser et al., from the 2016 KID sample, we assigned these into ranks from most common (1–9) to least common (≥500) to better highlight visual trends across hospital types and facilitate cross‐group comparison.^1^ This ranking method clarified relative differences in condition frequency, supporting clearer visualization of prevalence overall and by hospital type. We also identified conditions with the highest mean cost per encounter. As this approach identified uncommon conditions, these were not stratified into subgroups but were presented as summary results. Using all three KID datasets, we described temporal trends for the most common and most costly PECCS diagnosis groups for the 20 most common diagnoses. We additionally explored these trends based on hospital location.

All analyses were performed using the *survey* package (4.2‐1)^1^ in R, version 4.3.2 (R Foundation for Statistical Computing, Vienna, Austria). We reported weighted counts, proportions, and costs. For total volumes, we additionally expressed our findings with 95% confidence interval (CI).

## RESULTS

### Inclusion

After applying exclusions and survey weights, we identified an estimated 1,594,549 nonbirth pediatric hospitalizations that occurred in the United States in 2022. The median age was 5 years (interquartile range [IQR]: 0–13 years), and 51.0% were boys. Most hospitalizations occurred in urban teaching hospitals (58.7%), followed by freestanding children's hospitals (34.4%), urban nonteaching hospitals (3.8%), and rural hospitals (3.1%). A slight majority of admissions were covered by Medicaid (53.9%), followed by private insurance (38.5%). Medical complexity was present in 33.5% of admitted children, which was lower in rural (12.3%) and urban nonteaching (9.6%) hospitals and higher in urban teaching (29.2%) and freestanding children's hospitals (45.5%). The most common types of medical complexity were gastrointestinal (8.7% of all patients) and neuromuscular (8.6%) conditions. Demographics of the study sample, both overall and stratified by hospital location, are provided in Table 1.

**Table 1:** Characteristics of US pediatric hospitalizations in 2022; overall and stratified by hospital location.

Encounter characteristic	Overall	Rural	Urban non‐teaching	Urban teaching	Pediatric hospital
Total *N* (95% confidence interval)	1,594,549 (1,448,576, 1,740,522)	49,399 (40,867, 57,931)	59,884 (50,403, 69,366)	936,205 (845,522, 1,026,887)	549,061 (435,386, 662,736)
Age, years					
<1	405,877 (25.5)	13,192 (26.7)	16,813 (28.1)	230,305 (24.6)	145,568 (26.5)
1–4	369,170 (23.2)	9732 (19.7)	10,528 (17.6)	210,640 (22.5)	138,270 (25.2)
5–12	379,837 (23.8)	8182 (16.6)	10,706 (17.9)	213,649 (22.8)	147,300 (26.8)
13–17	439,664 (27.6)	18,293 (37.0)	21,838 (36.5)	281,611 (30.1)	117,923 (21.5)
Male sex	813,954 (51.0)	21,956 (44.4)	27,374 (45.7)	472,463 (50.5)	292,160 (53.2)
Race and ethnicity					
Native American	17,576 (1.1)	2072 (4.2)	661 (1.1)	10,398 (1.1)	4385 (0.8)
Asian or Pacific Islander	58,881 (3.7)	930 (1.9)	2021 (3.4)	34,530 (3.7)	21,446 (3.9)
Black	275,058 (17.2)	4881 (9.9)	6447 (10.8)	174,377 (18.6)	88,967 (16.2)
Hispanic	384,803 (24.1)	5384 (10.9)	16,521 (27.6)	214,698 (22.9)	148,967 (27.1)
White	760,204 (47.7)	34,839 (70.5)	30,537 (51.0)	438,596 (46.8)	256,154 (46.7)
Other	98,028 (6.1)	1293 (2.6)	3698 (6.2)	63,605 (6.8)	29,143 (5.3)
Payor status					
Medicare	3586 (0.2)	118 (0.2)	165 (0.3)	2,276 (0.2)	1027 (0.2)
Medicaid	859,714 (53.9)	30,296 (61.3)	33,654 (56.2)	512,437 (54.7)	283,324 (51.6)
Private insurance	614,198 (38.5)	16,058 (32.5)	22,440 (37.5)	361,112 (38.6)	214,584 (39.1)
Self‐pay	45,618 (2.9)	1630 (3.3)	1661 (2.8)	24,909 (2.7)	17,421 (3.2)
No charge	1464 (0.1)	Omitted^a^	52 (0.1)	1,041 (0.1)	363 (0.1)
Other	69,970 (4.4)	1,290 (2.6)	1912 (3.2)	34,430 (3.7)	32,344 (5.9)
Hospital region					
Northeast	274,844 (17.2)	9091 (18.4)	2440 (4.1)	204,062 (21.8)	59,250 (10.8)
Midwest	363,542 (22.8)	14,706 (29.8)	13,504 (22.5)	197,968 (21.1)	137,364 (25.0)
South	608,858 (38.2)	15,544 (31.5)	23,897 (39.9)	376,003 (40.2)	193,415 (35.2)
West	347,305 (21.8)	10,057 (20.4)	20,044 (33.5)	158,171 (16.9)	159,033 (29.0)
Emergency department visit associated with encounter	922,280 (57.8)	25,440 (51.5)	32,313 (54.0)	542,920 (58.0)	321,607 (58.6)
Transferred in from another acute care hospital	312,422 (19.6)	3626 (7.3)	8638 (14.4)	191,974 (20.5)	108,210 (19.7)
Injury code associated with encounter	159,145 (10.0)	3957 (8.0)	5058 (8.4)	105,048 (11.2)	45,082 (8.2)
Any medical complexity present	534,696 (33.5)	6078 (12.3)	5745 (9.6)	273,005 (29.2)	249,867 (45.5)
Type of medical complexity					
Cardiovascular	124,531 (7.8)	1109 (2.2)	1000 (1.7)	58,517 (6.3)	63,905 (11.6)
Congenital/genetic	73,272 (4.6)	859 (1.7)	722 (1.2)	33,882 (3.6)	37,810 (6.9)
Gastrointestinal	138,790 (8.7)	1349 (2.7)	1200 (2.0)	67,295 (7.2)	68,946 (12.6)
Hematologic/immunologic	117,597 (7.4)	841 (1.7)	638 (1.1)	57,607 (6.2)	58,510 (10.7)
Metabolic	103,800 (6.5)	1433 (2.9)	1347 (2.2)	56,467 (6.0)	44,552 (8.1)
Neonatal	46,168 (2.9)	548 (1.1)	709 (1.2)	22,203 (2.4)	22,707 (4.1)
Neuromuscular	137,685 (8.6)	1439 (2.9)	1,000 (1.7)	65,651 (7.0)	69,595 (12.7)
Renal	60,599 (3.8)	483 (1.0)	443 (0.7)	28,917 (3.1)	30,755 (5.6)
Respiratory	62,192 (3.9)	552 (1.1)	527 (0.9)	29,232 (3.1)	31,880 (5.8)
Transplant	19,988 (1.3)	1375 (2.8)	896 (1.5)	74,515 (8.0)	86,366 (15.7)
Malignancy	93,090 (5.8)	569 (1.2)	142 (0.2)	43,906 (4.7)	48,472 (8.8)
Disposition					
Routine discharge	1,485,370 (93.2)	45,193 (91.5)	55,369 (92.5)	866,903 (92.6)	517,905 (94.3)
Transfer to short‐term hospital	29,501 (1.9)	2477 (5.0)	2526 (4.2)	18,784 (2.0)	5,717 (1.0)
Transfer to other facility	35,122 (2.2)	917 (1.9)	1224 (2.0)	22,627 (2.4)	10,353 (1.9)
Home health care	31,977 (2.0)	527 (1.1)	368 (0.6)	20,943 (2.2)	10,136 (1.8)
Against medical advice	2920 (0.2)	136 (0.3)	256 (0.4)	1935 (0.2)	593 (0.1)

*Note*: Numbers represent *N* (%).

^a^ Omitted due to too few encounters, in accordance with the disclosure policies from the Healthcare Cost and Utilization Project.

### Most common diagnoses

**Figure 1:**
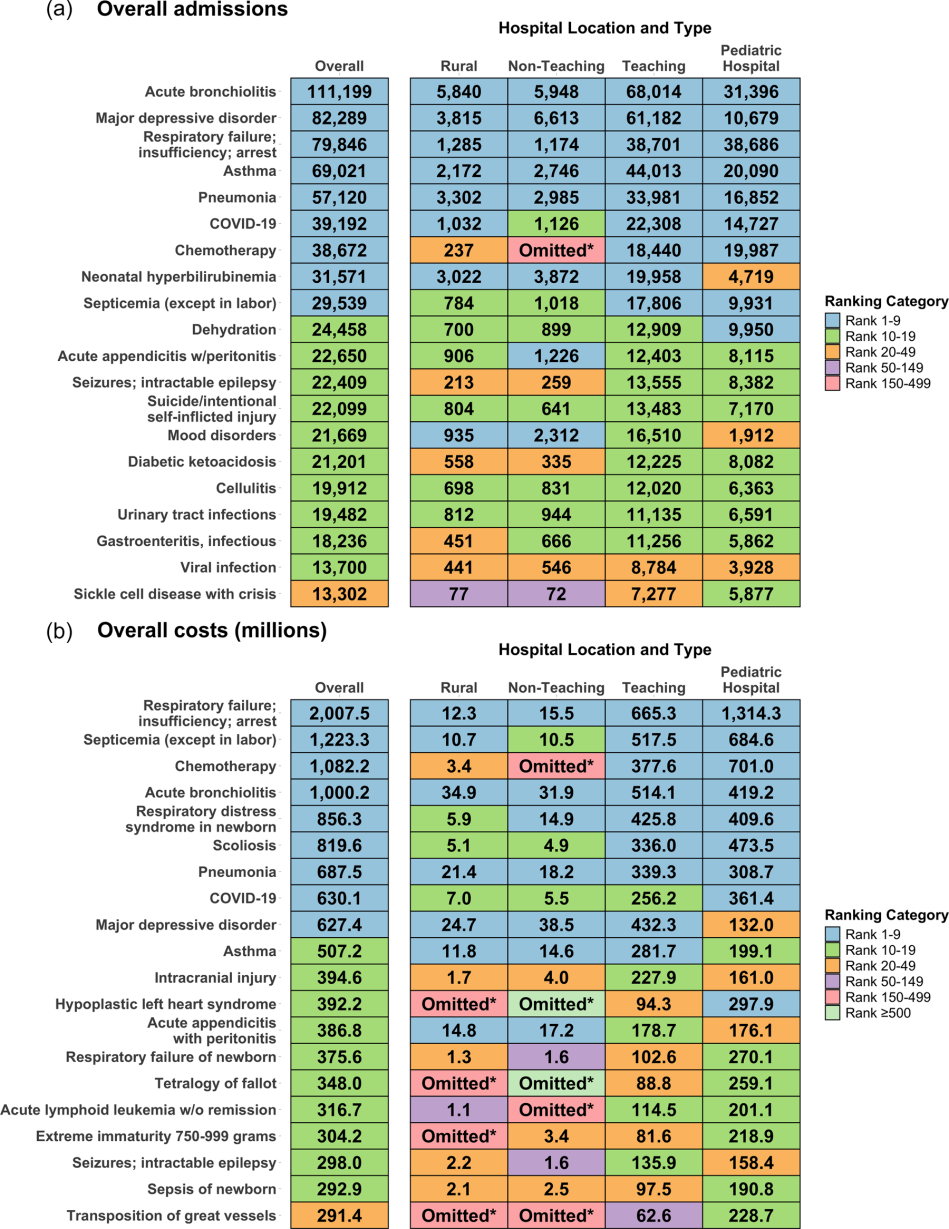
(a) Most common and (b) most costly diagnoses (in millions of dollars) for pediatric hospitalizations, overall and by hospital type. *Omitted due to too few encounters, in accordance with the disclosure policies from the Healthcare Cost and Utilization Project. **Mood disorders other than major depressive disorder, manic episodes, and major depressive disorder.

In the 2022 KID sample, the most common diagnoses were bronchiolitis (7.0%), major depressive disorder (5.2%), and respiratory failure (5.0%; Figure 1a). Mental health conditions (major depressive disorder, suicide and intentional self‐inflicted injury, and mood disorders) accounted for 3 of the 19 diagnoses comprising Ranks 1 and 2 for diagnosis frequency. When stratified by hospital type, major depressive disorder was ranked higher in urban nonteaching hospitals. In contrast, conditions of respiratory failure and chemotherapy ranked higher in freestanding children's hospitals.

### Costs

Overall, pediatric hospitalizations in 2022 accounted for $36.3 billion in costs. Ranked in order of costs, total costs from children's hospitals were the highest ($20.9 billion), followed by urban teaching hospitals ($14.5 billion), urban nonteaching hospitals ($0.47 billion), and rural hospitals ($0.39 billion). When evaluating rankings, the nine most costly conditions comprising Rank 1 accounted for 24.6% of total hospitalization costs for children. The costliest diagnoses overall were respiratory failure ($2.0B), septicemia ($1.2B), and chemotherapy ($1.1B; Figure 1b). Rare but expensive diagnoses, such as for conjoined twins, myeloid leukemia, and perinatal intestinal perforation, had the highest mean cost per patient (Table S1).

#### Longitudinal trends

Pediatric hospitalizations declined from 1,780,655 (95% CI: 1,629,010–1,932,300) in 2016 to 1,693,974 (95% CI: 1,547,562–1,840,385) in 2019 and 1,594,549 (95% CI: 1,448,576–1,740,522) in 2022, representing an overall 10.5% reduction from 2016 to 2022. Emergency department‐associated encounters increased from 50.5% in 2016 to 57.8% in 2022, while transfers from other hospitals increased from 14.6% to 19.6%. Hospitalizations became increasingly concentrated in urban and teaching settings: rural hospitals represented 4.5% of admissions in 2016, 3.5% in 2019, and 3.1% in 2022, with corresponding increases in admissions to urban teaching hospitals and freestanding children's hospitals. Urban teaching hospitals accounted for most encounters (approximately 54.7% in 2016 and 58.7% in 2022%), while freestanding children's hospitals contributed 31.1%‐34.4% (Table S2). During the 7‐year period, inflation‐adjusted costs rose from $32.1B in 2016 to $33.5B in 2019 and $35.9B in 2022.

**Figure 2:**
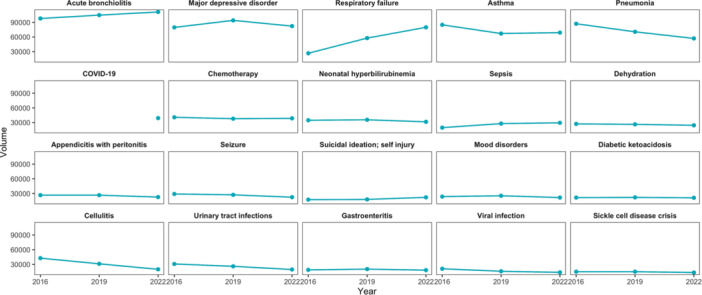
National trends in hospitalization volume for the most common pediatric diagnoses across 2016, 2019, and 2022.

**Figure 3:**
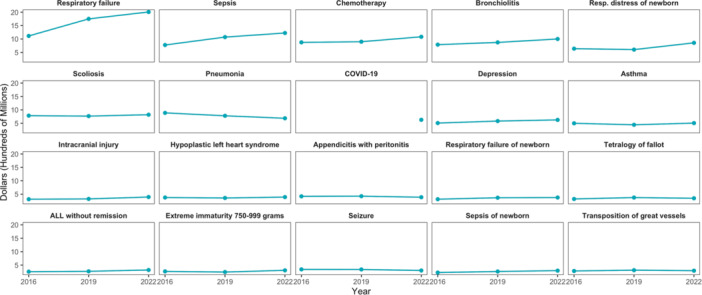
National trends in inpatient costs for the most common pediatric diagnoses across 2016, 2019, and 2022.

Across the 3 study years, volume and cost patterns by diagnosis revealed evolving trends in pediatric inpatient care. Some respiratory conditions demonstrated a consistent rise, including for bronchiolitis and respiratory failure, whereas those for pneumonia and asthma declined (Figure 2). Mental health diagnoses demonstrated less consistent changes over time. Some conditions, such as major depression, decreased in 2022 relative to 2019, whereas others did not change substantially. Stratified by hospital type, some location‐specific patterns were notable. For example, admissions for acute bronchiolitis increased in urban teaching hospitals, and admissions for major depressive disorder declined slightly in urban nonteaching hospitals (Figure S1). Considering costs over time, respiratory failure, sepsis, and chemotherapy remained the leading contributors to inpatient costs (Figure 3); these persisted when stratified based on hospital location (Figure S2).

## DISCUSSION

We identified common and costly US pediatric nonbirth hospitalizations from nationally representative data from 2022, with additional longitudinal analysis of data from 2016 to 2019. Overall, we observed a 10.5% decline in total admissions during the 7‐year time frame. Compared with prior analyses,^1^ we identified a rise in the relative importance of mental health conditions, particularly major depressive disorder and suicidality, compared with similar analyses performed in prior years.^1^, ^5^ These findings highlight a shifting landscape in pediatric inpatient care, with mental health presentations occupying a growing share of inpatient bedspace, though respiratory conditions continue to utilize substantial resources.

The KID provides a large, nationally representative sample across diverse hospital types, allowing us to assess hospitalization trends with broad generalizability. The stratified framework used in the present analysis helps identify conditions that may be underappreciated in national aggregates but are highly impactful within specific hospital settings. These results thus reinforce recent reports that youth mental health crises are increasingly contributing to hospital utilization and resource allocation nationwide.^12^ Admissions for major depressive disorder, outpacing pneumonia and asthma, declined in total counts in 2022 as compared with 2019, though admissions for suicidality increased. While broader shifts in billing and coding practices may also be partially contributing to these changes^12^ these findings corroborate with other literature pertaining to an ongoing rise in mental health issues in youth, which continued to rise following the pandemic. Specifically, multicenter data have demonstrated increased pediatric hospitalizations for mental health conditions^13^, ^14^ as well as rising mental health emergency visits across the United States.^15^ This finding is likely thus reflective of the rising frequency and acuity of these presentations over time. To address the growth of mental healthcare needs among admitted youth, a multipronged approach has been advocated. Hospital‐based interventions to address increased healthcare use for suicidality may include the delivery of brief interventions in emergency department settings,^16^ such as safety planning, and improved linkage to follow‐up care.

Our findings suggest that pediatric care continues a trend toward greater concentration in urban and teaching hospitals, with declining admissions in rural and nonteaching centers. In our analysis, rural hospitals accounted for 3.1% of total admissions in 2022, down from 4.5% in 2016 and 3.5% in 2019. Nonteaching hospitals represented 3.8% of admissions, reflecting a decline from 9.7% in 2016 and 5.2% in 2019. These findings may stem from lower reimbursement rates and staffing shortages for rural inpatient pediatric care, leading to further capacity reductions and loss of regional access. Given projected decreases in Medicaid funding, these decreasing admissions highlight the need for nonfederal solutions including regional training, telehealth support, bundled pediatric service models, and state policies that tie hospital funding to community accountability and pediatric readiness.^5^, ^17^, ^18^

Our findings emphasize the importance of research and policy activities focused on improving pediatric mental health and maintaining equitable geographic access to pediatric inpatient services, particularly for children in rural communities where pediatric inpatient services continue to decline. While this analysis highlights a rise of mental health conditions requiring admission, respiratory conditions still played a dominant role, with respect to both overall volumes and costs. This pattern aligns with trends from the literature describing the 2022 “tripledemic” of respiratory syncytial virus, influenza, and severe acute respiratory syndrome coronavirus 2 (SARS‐CoV‐2) that led to prolonged and heterogeneous surges in pediatric emergency and inpatient volumes across US children's hospitals, straining already limited capacity despite stable national averages.^2^ Further analysis is warranted to assess whether the increase in respiratory failure reflects shifts in coding practices or changes in severity.

Beyond findings related to inpatient volume, our analysis also evaluated costs associated with pediatric admissions. After adjusting for inflation, most condition‐specific costs remained relatively stable across the study period; however, expenditures related to respiratory failure, which have historically represented the highest cost diagnosis among pediatric inpatients, continued to rise, with greater growth among urban teaching and freestanding children's hospitals. The persistently high and increasing cost burden of respiratory conditions aligns with prior analyses of children's hospitals, underscoring the disproportionate resource demands of critical respiratory care.^19^, ^20^ Given that most pediatric hospitalizations are covered by Medicaid,^18^ rising costs concentrated in freestanding children's hospitals may threaten their financial stability, particularly in the context of constrained public reimbursement rates. Sustaining equitable access to pediatric inpatient care in the face of growing regionalization will therefore require models that account for the complexity and intensity of care provided in these higher acuity settings.

Our findings are subject to limitations. The KID is a discharge‐level database and does not track individual patients, so repeated admissions by the same patient may overestimate hospitalization burden. Diagnoses and procedures are identified using ICD‐10‐CM billing codes, which are subject to coding variation, misclassification, and shifting documentation practices over time. The data set also lacks certain clinical details, such as laboratory values or in‐hospital treatments, that may better characterize disease severity or quality of care. Race and ethnicity data are reported by individual hospitals and may be incomplete or inconsistently categorized, limiting assessment of disparities. Furthermore, we were unable to assess observation stays or outpatient visits, which may underestimate the total healthcare use of certain conditions. Finally, our cost estimates are derived from hospital‐reported charges and cost‐to‐charge ratios, which may not reflect actual resource utilization or societal costs. When using the KID database across years, one limitation is that changes in variable definitions, coding schemes, and data collection methods can affect comparability and consistency of results over time.

## CONCLUSION

Between 2016 and 2022, pediatric hospitalizations in the United States declined by more than 10%, reflecting both a sustained reduction in inpatient capacity and a shifting diagnostic landscape. Admissions for mental health conditions increased in rank, though respiratory conditions continued to be an important contributor toward resource use. These findings underscore the need for strengthened pediatric mental health infrastructure, regional coordination networks, and sustainable models that support rural and community hospitals. Policymakers and health systems should prioritize investments that enhance pediatric readiness, expand telehealth partnerships, and address high‐burden conditions to promote equitable, resilient, and accessible inpatient care for children nationwide.

## CONFLICT OF INTEREST STATEMENT

The authors declare no conflict of interest.

## ETHICS STATEMENT

This study was approved by the Ann & Robert H. Lurie Children's Hospital Institutional Review Board (STUDY00000079).

## Supporting information

Supplementary Figure 1. National trends in hospitalization volume for the most common pediatric diagnoses across 2016, 2019, and 2022, stratified by hospital type.

Supplementary Figure 2. National trends in inpatient cost for the most common pediatric diagnoses across 2016, 2019, and 2022, stratified by hospital type.

Supplementary_Table_1.

Supplementary_Table_2.

## Data Availability

The data used in this study are made available by the Healthcare Cost and Utilization Project.
