# Supplementary figures and images for: Prevalence and costs of US pediatric hospitalizations, 2022
Source: J Hosp Med. 2026 Feb 10;21(8):872–80. doi: 10.1002/jhm.70272 (PMC13050237; doi:10.1002/jhm.70272)

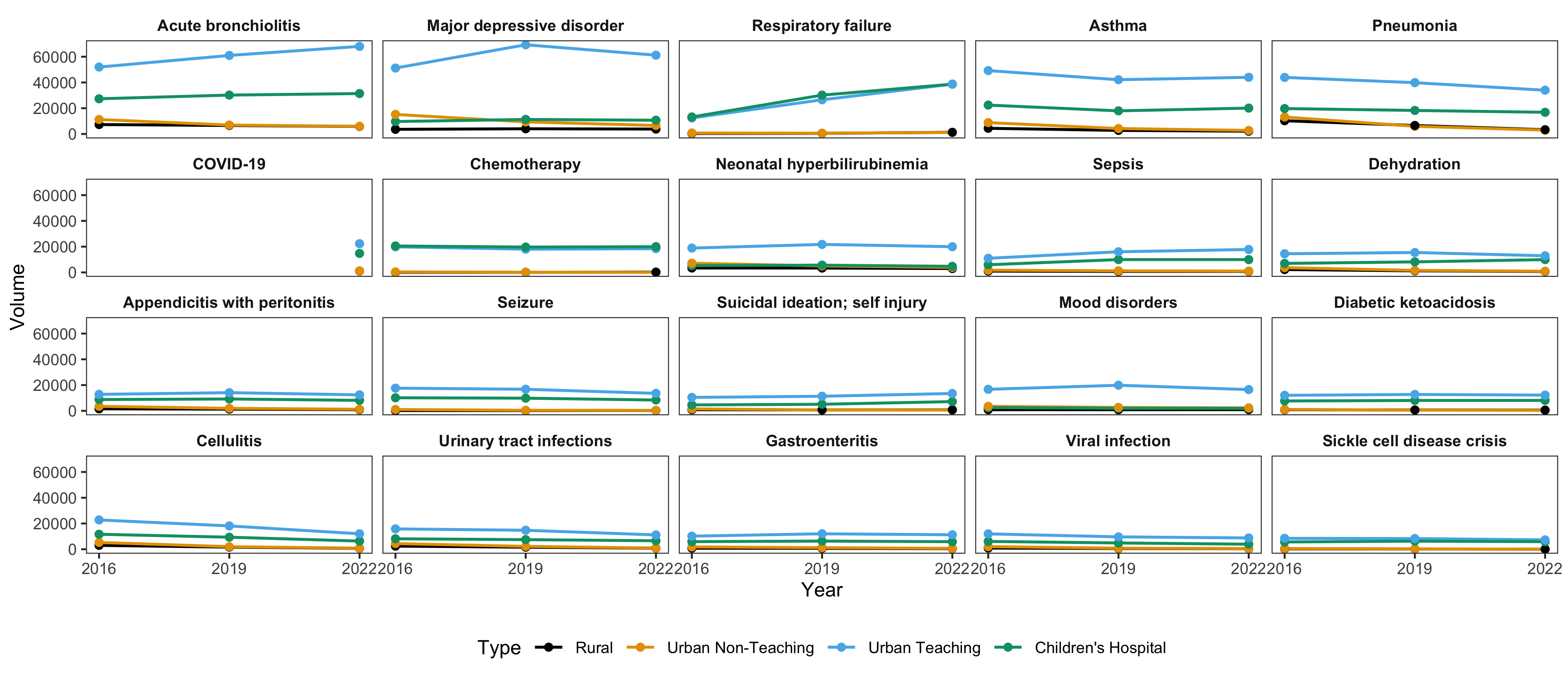

Supplement: Supplementary file 1 — Supplementary Figure 1. National trends in hospitalization volume for the most common pediatric diagnoses across 2016, 2019, and 2022, stratified by hospital type. [file JHM-21-872-s002.tif]

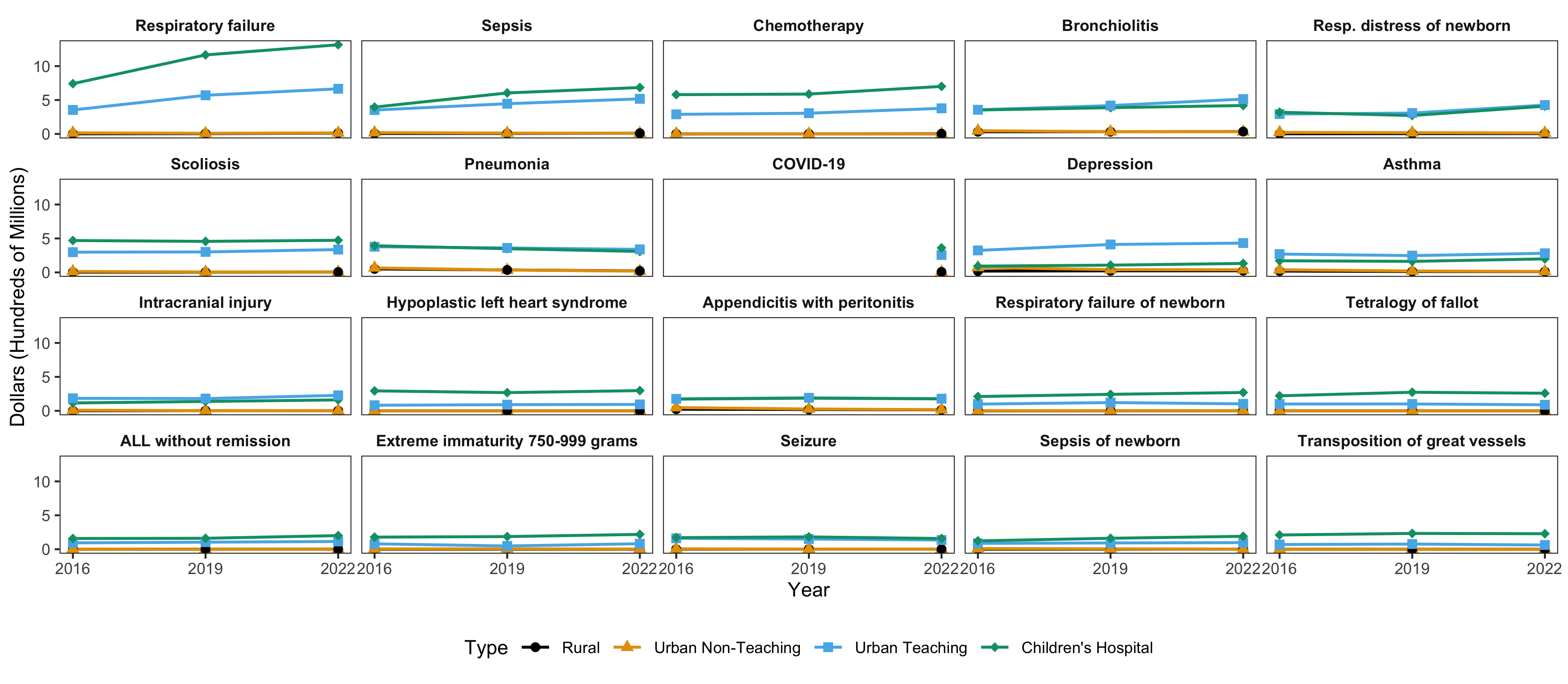

Supplement: Supplementary file 2 — Supplementary Figure 2. National trends in inpatient cost for the most common pediatric diagnoses across 2016, 2019, and 2022, stratified by hospital type. [file JHM-21-872-s004.tif]
