# Supplementary material for: Prevalence and costs of US pediatric hospitalizations, 2022
Source: J Hosp Med. 2026 Feb 10;21(8):872–80. doi: 10.1002/jhm.70272 (PMC13050237; doi:10.1002/jhm.70272)
Supplement: Supplementary file 3 — Supplementary_Table_1. [file JHM-21-872-s003.docx]

**Supplementary Table 1.** Conditions with the highest average cost per encounter, ranked in descending order.

| **Diagnosis** | **Total cost (millions)** | **Total count (thousands)** | **Mean cost per encounter (thousands of dollars)** |
| --- | --- | --- | --- |
| Conjoined twins | Omitted* | Omitted* | Omitted* |
| Chronic myeloid leukemia in relapse | Omitted* | Omitted* | Omitted* |
| Myeloid leukemia | 4.7 | 0.01 | 425.8 |
| Perinatal intestinal perforation | 120.5 | 0.32 | 380.6 |
| Extreme immaturity 500-749 grams | 288.8 | 0.84 | 344.4 |
| Acute myeloid leukemia in remission | 43.5 | 0.13 | 342.5 |
| Bronchopulmonary dysplasia | 262.6 | 0.81 | 322.5 |
| Chronic myeloid leukemia in remission | Omitted* | Omitted* | Omitted* |
| Congenital and developmental myasthenia | 5.83 | 0.02 | 310.7 |
| Extreme immaturity 750-999 grams | 304.3 | 1.01 | 302.6 |
| Acute megakaryoblastic leukemia | 12.5 | 0.04 | 291.6 |
| Hydrops fetalis not due to isoimmunization | 19.2 | 0.07 | 280.6 |
| Extreme immaturity <500 grams | 39.9 | 0.14 | 276.0 |
| Myelodysplastic disease | 4.8 | 0.02 | 275.3 |
| Congenital myopathies | 9.0 | 0.03 | 273.1 |
| Combined immunity deficiency | 22.7 | 0.08 | 272.0 |
| Bulbus cordis anomalies and anomalies of cardiac septal closure, other | 47.8 | 0.18 | 262.4 |
| Hypoplastic left heart syndrome | 392.2 | 1.50 | 261.4 |
| Anomalies of diaphragm, congenital | 190.2 | 0.78 | 244.6 |
| Acute myeloid leukemia in relapse | 59.4 | 0.25 | 237.8 |

^*^Omitted due to too few encounters, in accordance with the disclosure policies from the Healthcare Cost and Utilization Project.
