# Supplementary material for: Prevalence and costs of US pediatric hospitalizations, 2022
Source: J Hosp Med. 2026 Feb 10;21(8):872–80. doi: 10.1002/jhm.70272 (PMC13050237; doi:10.1002/jhm.70272)
Supplement: Supplementary file 4 — Supplementary_Table_2. [file JHM-21-872-s001.docx]

**Supplementary Table 2.** Overall characteristics of included encounters, by study year. Numbers represent N (%).

| **Encounter Characteristic** | **2016** | **2019** | **2022** |
| --- | --- | --- | --- |
| **Total N (95% Confidence Interval)** | **1,780,655 (1,629,010, 1,932,300)** | **1,693,974 (1,547,562, 1,840,385)** | **1,594,549 (1,448,576, 1,740,522)** |
| **Age, years** |  |  |  |
| <1 | 451,482 (25.4) | 431,845 (25.5) | 405,877 (25.5) |
| 1-4 | 379,834 (21.3) | 368,058 (21.7) | 369,170 (23.2) |
| 5-12 | 451,167 (25.3) | 423,563 (25.0) | 379,837 (23.8) |
| 13-17 | 498,172 (28.0) | 470,507 (27.8) | 439,664 (27.6) |
| **Male sex** | 911,576 (51.2) | 870,112 (51.4) | 813,954 (51.0) |
| **Race and ethnicity** |  |  |  |
| Native American | 18,398 (1.0) | 17,849 (1.1) | 17,576 (1.1) |
| Asian or Pacific Islander | 59,931 (3.4) | 60,541 (3.6) | 58,881 (3.7) |
| Black | 314,263 (17.6) | 296,836 (17.5) | 275,058 (17.2) |
| Hispanic | 409,242 (23.0) | 391,829 (23.1) | 384,803 (24.1) |
| White | 880,504 (49.4) | 824,713 (48.7) | 760,204 (47.7) |
| Other | 98,316 (5.5) | 102,206 (6.0) | 98,028 (6.1) |
| **Payor status** |  |  |  |
| Medicare | 6,251 (0.4) | 4,820 (0.3) | 3,586 (0.2) |
| Medicaid | 977,136 (54.9) | 927,839 (54.8) | 859,714 (53.9) |
| Private insurance | 681,453 (38.3) | 646,839 (38.2) | 614,198 (38.5) |
| Self-pay | 44,090 (2.5) | 49,721 (2.9) | 45,618 (2.9) |
| No charge | 1,252 (0.1) | 1,980 (0.1) | 1,464 (0.1) |
| Other | 70,473 (4.0) | 62,774 (3.7) | 69,970 (4.4) |
| **Hospital region** |  |  |  |
| Northeast | 320,249 (18.0) | 284,277 (16.8) | 274,844 (17.2) |
| Midwest | 389,442 (21.9) | 377,030 (22.3) | 363,542 (22.8) |
| South | 685,626 (38.5) | 651,795 (38.5) | 608,858 (38.2) |
| West | 385,338 (21.6) | 380,872 (22.5) | 347,305 (21.8) |
| **Emergency department visit associated with encounter** | 898,966 (50.5) | 918,697 (54.2) | 922,280 (57.8) |
| **Transferred in from another acute care hospital** | 260,673 (14.6) | 313,996 (18.5) | 312,422 (19.6) |
| **Injury code associated with encounter** | Not available in this dataset | 159,431 (9.4) | 159,145 (10.0) |
| **Any medical complexity present** | 559,160 (31.4) | 560,073 (33.1) | 534,696 (33.5) |
| **Type of medical complexity** |  |  |  |
| Cardiovascular | 114,926 (6.5) | 123,734 (7.3) | 124,531 (7.8) |
| Congenital/genetic | 73,643 (4.1) | 77,414 (4.6) | 73,272 (4.6) |
| Gastrointestinal | 130,135 (7.3) | 142,408 (8.4) | 138,790 (8.7) |
| Hematologic/immunologic | 90,489 (5.1) | 87,604 (5.2) | 117,597 (7.4) |
| Metabolic | 91,874 (5.2) | 96,614 (5.7) | 103,800 (6.5) |
| Neonatal | 36,014 (2.0) | 44,479 (2.6) | 46,168 (2.9) |
| Neuromuscular | 141,038 (7.9) | 146,450 (8.6) | 137,685 (8.6) |
| Renal | 56,210 (3.2) | 61,244 (3.6) | 60,599 (3.8) |
| Respiratory | 61,060 (3.4) | 67,436 (4.0) | 62,192 (3.9) |
| Transplant | 22,330 (1.3) | 22,103 (1.3) | 19,988 (1.3) |
| Malignancy | 147,982 (8.3) | 100,056 (5.9) | 93,090 (5.8) |
| **Disposition** |  |  |  |
| Routine discharge | 1,652,034 (92.8) | 1,573,295 (92.9) | 1,485,370 (93.2) |
| Transfer to short-term hospital | 32,081 (1.8) | 31,765 (1.9) | 29,501 (1.9) |
| Transfer to other facility | 34,108 (1.9) | 34,144 (2.0) | 35,122 (2.2) |
| Home health care | 49,622 (2.8) | 42,961 (2.5) | 31,977 (2.0) |
| Against medical advice | 2,718 (0.2) | 2,671 (0.2) | 2,920 (0.2) |
| **Hospital Location** |  |  |  |
| Rural | 81,248 (4.5) | 60,114 (3.5) | 49,399 (3.1) |
| Urban non-teaching | 174,818 (9.7) | 88,655 (5.2) | 59,884 (3.8) |
| Urban teaching | 985,995 (54.7) | 984,812 (58.1) | 936,205 (58.7) |
| Pediatric hospital | 560,525 (31.1) | 560,392 (33.1) | 549,061 (34.4) |

*Omitted due to too few encounters, in accordance with disclosure policies from the Healthcare Cost and Utilization Project.
